# Retraction Note: Combination of sorafenib and gadolinium chloride (GdCl3) attenuates dimethylnitrosamine(DMN)-induced liver fibrosis in rats

**DOI:** 10.1186/s12876-022-02495-4

**Published:** 2022-09-15

**Authors:** Cheng Liu, Zongguo Yang, Lei Wang, Yunfei Lu, Bozong Tang, Hui Miao, Qingnian Xu, Xiaorong Chen

**Affiliations:** 1 Department of Traditional Chinese Medicine, Shanghai Public Health Clinical Center, Shanghai, 201508 China; 2 Laboratory of Molecular Pathology, Central Laboratory, Putuo Hospital, Shanghai University of Traditional Chinese Medicine, Shanghai, 200062 China; 3 Department of Hepatology, Affiliated Hospital of Shandong University of Traditional Chinese Medicine, Jinan, 250014 China

## Retraction Note: BMC Gastroenterology (2015) 15:159 10.1186/s12876-015-0380-5

The Editor has retracted this article. After publication, concerns were raised regarding western blot image similarities between Fig. 3C in this article and Fig. 3B in another article with one shared author [1] that was under consideration within a close time frame.

Additionally, Figs. 2 and 4 appear to have breaks in the western blot backgrounds. The authors have confirmed that some of the images were composed from different experiments.

The authors have been unable to provide the original blots from the experiments reported in this article. The Editor therefore no longer has confidence in the presented data.

None of the authors have responded to any correspondence from the editor or publisher about this retraction notice.
